# Development of Biopredictive Dissolution Method for Extended-Release Desvenlafaxine Tablets

**DOI:** 10.3390/pharmaceutics15051544

**Published:** 2023-05-19

**Authors:** Gustavo Vaiano Carapeto, Marcelo Dutra Duque, Michele Georges Issa, Humberto Gomes Ferraz

**Affiliations:** 1 Department of Pharmacy, Faculty of Pharmaceutical Sciences, Universidade de São Paulo—USP, Av. Prof. Lineu Prestes 580, São Paulo 05508-080, SP, Brazil; sferraz@usp.br; 2 Department of Pharmaceutical Sciences, Institute of Environmental, Chemical and Pharmaceutical Sciences, Universidade Federal de São Paulo—UNIFESP, Rua São Nicolau, 210 Centro, Diadema 09913-030, SP, Brazil; marcelo.duque@unifesp.br

**Keywords:** PBBM, DoE, desvenlafaxine, biopredictive dissolution method, Taguchi

## Abstract

This study aimed to develop a biopredictive dissolution method for desvenlafaxine ER tablets using design of experiments (DoE) and physiologically based biopharmaceutics modeling (PBBM) to address the challenge of developing generic drug products by reducing the risk of product failure in pivotal bioequivalence studies. For this purpose, a PBBM was developed in GastroPlus^®^ and combined with a Taguchi L9 design, to evaluate the impact of different drug products (Reference, Generic #1 and Generic #2) and dissolution test conditions on desvenlafaxine release. The influence of the superficial area/volume ratio (SA/V) of the tablets was observed, mainly for Generic #1, which presented higher SA/V than the others, and a high amount of drug dissolved under similar test conditions. The dissolution test conditions of 900 mL of 0.9% NaCl and paddle at 50 rpm with sinker showed to be biopredictive, as it was possible to demonstrate virtual bioequivalence for all products, despite their release-pattern differences, including Generic #3 as an external validation. This approach led to a rational development of a biopredictive dissolution method for desvenlafaxine ER tablets, providing knowledge that may help the process of drug product and dissolution method development.

## 1. Introduction

A major concern surrounding the development of generic drug products are the bioequivalence studies, which require time and resource investments. Therefore, pharmaceutical companies must work on formulation candidates and dissolution methods to reduce the risk of a product to fail on a pivotal bioequivalence study. In this sense, the development of biopredictive in vitro dissolution methods for testing generic drug products would lead to both a higher rational development and a reduction in the risk of failing on bioequivalence studies [1].

Physiologically based biopharmaceutics modeling (PBBM) is used as an in silico tool that integrates in vitro dissolution data to pharmacokinetic and absorption models. It can be used to predict the in vivo drug behavior from dissolution data and to support the development and assessment of biopredictive dissolution methods, reducing the need for conducting in vivo studies [2].

Approved in 2008 for the treatment of major depressive disorder by the Food and Drug Administration [3], desvenlafaxine is a class I drug according to the Biopharmaceutics Classification System (BCS) [4]. Desvenlafaxine is available in its succinate salt form and is commercialized as extended-release (ER) tablets containing 50 mg and 100 mg of the drug [5].

Since drug release from desvenlafaxine tablets is dependent on the hydrophilic matrix, it is important to obtain in vitro dissolution test conditions that are adequate for the gel formation and consequent drug diffusion, avoiding the adherence of the tablet in the dissolution vessel and promoting appropriate dissolution hydrodynamics [6]. Therefore, if the dissolution method is not well designed, it may not represent the in vivo dissolution, increasing the risk of a developed product containing desvenlafaxine not being approved in bioequivalence studies [7].

In addition, a product monograph for desvenlafaxine ER tablets is not yet available in the United States Pharmacopeia [8]; therefore, a specific dissolution method is also not described. Furthermore, the FDA dissolution methods database provides a method recommending the use of USP apparatus 1 at 50 rpm and 900 mL of 0.9% NaCl in purified water as the dissolution medium [9].

Due to few information available on biopredictive dissolution methods for desvenlafaxine ER formulations, the present study aims to develop a biopredictive dissolution method for these tablets using design of experiments (DoE) and PBBM. This reflects in a rational design which is in line with the quality-by-design (QbD) approach, an important approach for the pharmaceutical industry [10].

## 2. Materials and Methods

### 2.1. Materials

The reference drug product for desvenlafaxine extended-release (ER) tablets in Brazil, Pristiq™ (produced by Pfizer Ireland Pharmaceuticals, and marketed and distributed in Brazil by Wyeth Indústria Farmacêutica Ltd.a), and three generic drug products (ER tablets containing desvenlafaxine) currently available in the Brazilian market were selected for this study. Pristiq™ and the generic drug products were denoted in the study as “Reference”, “Generic #1”, “Generic #2”, and “Generic #3”. All drug products analyzed were obtained and used within their expiration date.

The analytical reagents were used to prepare the solution for the dissolution tests and/or for drug quantification: sodium chloride, sodium hydroxide, and monobasic potassium phosphate (Casa Americana, São Paulo, Brazil).

Desvenlafaxine succinate, kindly provided by Aché Laboratórios Farmacêuticos S.A. (Guarulhos, São Paulo, Brazil), 97.8% purity degree, was used as work standard for drug quantification in the dissolution tests.

### 2.2. Integrated DoE and PBBM Workflow Diagram

**Figure 1:**
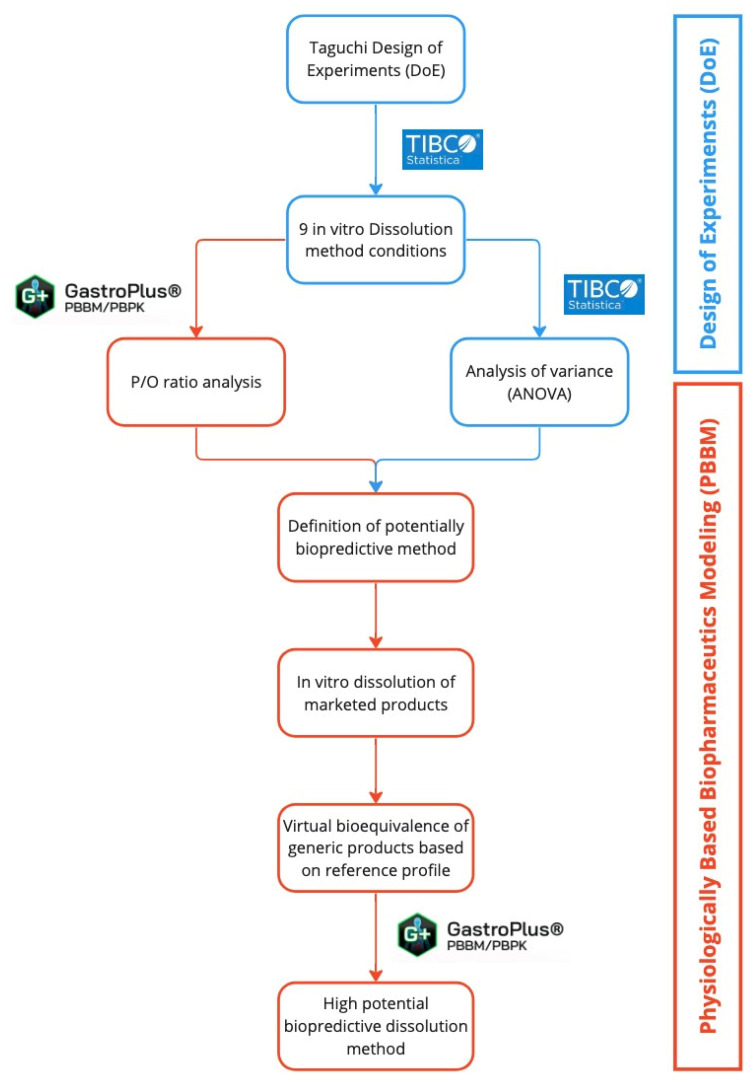
Diagram representing the workflow of the present study.

The work developed in the present study integrated the use of statistical design of experiments (DoE) with physiologically based biopharmaceutics modeling (PBBM), and it is represented in the diagram shown in Figure 1.

### 2.3. PBBM Development and Refinement

**Figure 2:**
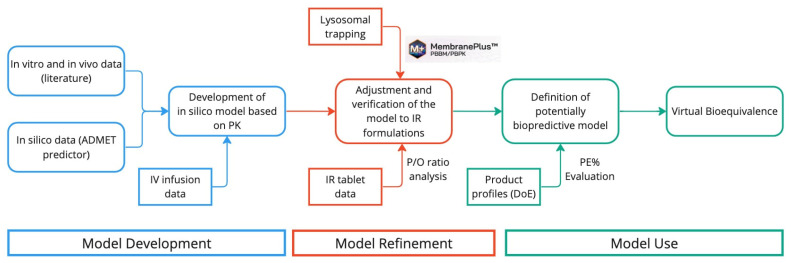
Diagram representing the workflow of the PBBM development and refinement for the final model to be used to run virtual bioequivalence studies.

The process of developing the PBBM and its refinement leading to the model used is described graphically in Figure 2.

Desvenlafaxine present pKa and logP values, which are within the values of compounds that are subjected to be trapped in the lysosomes. These values are basic pKa 6.5–11 and logP > 2 [11,12].

Additionally, desvenlafaxine presents high Tmax (~3–4 h) for a BCS class I drug in the IR dosage forms. Therefore, before model building in Gastroplus^®^, the fraction of drug unbound to the enterocytes was investigated using the MembranePlus™ version 2.0 software (Simulations Plus Inc., Lancaster, CA, USA).

Drug characteristics, described in Table 1, were used as input data in the software. The accumulation of desvenlafaxine in the lysosomes was evaluated by running Caco-2 permeability simulations in the software, considering the default pH of lysosomes (pH 4.0) and changing the pH to 6.5, using the in silico experimental conditions described by Bolger et al. [12].

A parameter sensitivity analysis was also performed in GastroPlus^®^ to evaluate the influence of the fraction unbound in the enterocytes (Fuent) on Tmax, and to support the selection of the value of Fuent to be included in the model.

A PBBM was developed using the GastroPlus^®^ software version 9.8.3 (Simulations Plus Inc., Lancaster, CA, USA). Desvenlafaxine physicochemical and biopharmaceutical properties were used as input data in the software (Table 1). Distribution and elimination of the drug were set up using a top-down approach, building a compartmental pharmacokinetic model based on intravenous administration of the drug to healthy male and females volunteers aged between 18–45 years, with an average age of 29.6 years old, with 81.7 kg of body weight and 24.6 km/m^2^ of body mass index [13], which was refined using data from the administration of immediate-release (IR) tablets to 35 volunteers (male and female) aged between 18–45 years old [4,14].

Simulations were performed considering the IR tablet as the dosage form containing 50 mg of the drug administered with 250 mL of water [4,14].

The model was verified by running a simulation and changing the dose for the IR tablet to 100 mg of the drug. The PK data obtained were compared to the literature data [4,14].

### 2.4. Design of Experiments (DoE) for the In Vitro Dissolution Methods

A Taguchi orthogonal array was used to assess which dissolution and formulation factors affect the release profile of desvenlafaxine ER tablets; thus, a 4-factor/3-level matrix design (Table 2) was created using the Statistica^®^ software version 13 (TIBCO Software Inc., Palo Alto, CA, USA), resulting in 9 runs (Table 3).

The data generated from these runs were statistically analyzed using the same software. This evaluation was based on the analysis of variance (ANOVA) of the responses: Q%2 h, Q%12 h, and Q%24 h.

### 2.5. Dissolution Tests

The dissolution tests described in Table 3 were performed using 900 mL of dissolution medium at 37 °C for each run with the dissolution equipment 708-DS (Agilent Technologies Inc., Santa Clara, CA, USA), with six units of each product. Dissolution media were prepared and degassed by heating at 41 °C, followed by vacuum filtration using 0.45 µm pore size cellulose acetate membrane.

For each dissolution test, aliquots of 5 mL were automatically collected using a VK 8000 Automatic Dissolution Sampling Station (Agilent Technologies Inc., Santa Clara, CA, USA) at 1, 2, 4, 8, 12, 16, 20, and 24 h. The samples were filtered using a 45 µm pore size polyethylene membrane during each aliquot collection.

The amount of desvenlafaxine dissolved was quantified by UV spectrophotometry, using a Thermo Scientific Evolution 201 spectrophotometer (Thermo Scientific, Oxford, UK) at 223.5 nm using quartz cuvette with a pathlength of 10 mm.

### 2.6. Physical Characterization of the Drug Products

Samples of the drug products Reference, Generic #1, Generic #2, and Generic #3 were submitted for the evaluation of tablet average weight, thickness, and diameter. For the evaluation of the tablet average weight, 12 units of each drug product were weighed on a Shimadzu^®^ model BL 3200H analytical balance (Shimadzu^®^ Corporation, Kyoto, Japan). Tablet dimensions (thickness and diameter/length) were manually evaluated using a digital caliper (Mitutoyo, Kanagawa, Japan) using *n* = 12 units of each drug product.

These dimensions were used to calculate the surface area/volume (SA/V) ratio of each drug product. Since the reference drug product is square shaped and the generic drug products are round, they require different equations to calculate surface area and volume. For calculation purposes, the tablet shapes were considered as either cylindrical or rectangular prisms. For the round-shaped tablets, a cylindrical surface area and volume equations were applied (Equations (1) and (2)), as for the square-shaped tablets, a rectangular prism surface area and volume equations were applied (Equations (3) and (4)) [15].

(1) $$SA\;cylinder=2\pi r\left(r+t\right),$$

(2) $$V\;cylinder=\pi r^{2}t,$$

(3) $$SA\;retangular\;prism=2l^{2}+4lt,$$

(4) $$V\;retangular\;prism=tl^{2}.$$

where r is the tablet radius, l is the tablet length, and t is the tablet thickness.

### 2.7. Selection of the Biopredictive Dissolution Method

The release profiles obtained from different dissolution test conditions as described in Table 3 were used as input data in the software. For each dissolution profile, a drug record was created in which the plasma concentration–time curve of the reference drug product Pristiq™ 50 mg ER tablets was used as an opd file, from a bioequivalence study conducted with 44 healthy volunteers aged between 18–50 years [16].

The IVIVCPlus™ module in GastroPlus^®^ was used to mechanistically deconvolute the plasma concentration–time curve of the reference drug product, obtaining the fraction in vivo released, which was correlated to each in vitro dissolution profile. The equations obtained were used in the convolution to evaluate which dissolution profile would generate the reconstructed plasma profile that best matches the observed plasma concentration curve.

### 2.8. Dissolution Evaluation of the Drug Products Using the Biopredictive Method

The drug products used in the DoE (Reference, Generic #1, and Generic #2) were submitted to the dissolution test using the biopredicted method selected based on the simulations. For an external evaluation, the drug product Generic #3, which was not used in the DoE, was also evaluated using the selected dissolution method.

The dissolution test conditions volume of dissolution medium, temperature, and sampling time points were the same as previously described (Section 2.5).

### 2.9. Virtual Bioequivalence Studies

Virtual bioequivalence (VBE) studies were run in GastroPlus^®^ using the population simulator. For this purpose, crossover VBE were run while comparing the drug products Generic #1, Generic #2, and Generic #3 with the Reference. The number of virtual subjects was 25 for each VBE, considering the fasted state, and 10 trials were run for each VBE.

The bioequivalence was considered if the lower and upper limits of the 90% confidence interval (CI) of the geometric mean ratios of C_max_ test/C_max_ reference and AUC_0-t_ test and AUC_0-t_ reference were within the 0.8–1.25 interval [17].

Based on the results of VBE, dissolution test specifications were proposed.

## 3. Results and Discussion

### 3.1. PBBM Development and Refinement

A pH-dependent permeability was observed and described for desvenlafaxine IR tablets [4]. Experimental values were incorporated by these authors in their model to explain a delay in the T_max_ of desvenlafaxine IR tablets.

However, in our model in GastroPlus^®^, this pH-dependent permeability is automatically considered by the software due to the absorption scale factors (ASF) in the Advanced Compartmental Absorption and Transit (ACAT™) model. The ASF uses the permeability (Peff) and the logD values of the drug, considering the differences in permeability that occur in the gastrointestinal tract due to the pH. Since it is already considered in the software, and the drug presents characteristics of a lysosomal tropic agent, we decided to evaluate it using the MembranePlus™ software.

**Figure 3:**
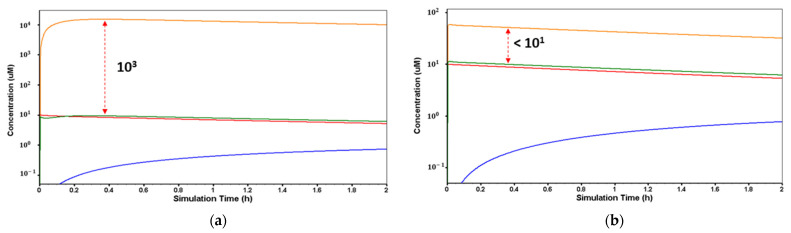
Simulated desvenlafaxine concentration in the lysosomes (orange), cytosol (green), donor compartment (red), receiver compartment (blue) from in silico Caco-2 permeability evaluation using MembranePlus™ software. (**a**) Lysosome normal pH = 4.0 and (**b**) lysosome altered pH = 6.5.

As it was expected, due to the pka and logP values of desvenlafaxine, the lysosomal trapping evaluation of the drug using the MembranePlus™ software showed that the drug concentration in the lysosomes at pH = 4.0 (Figure 3a) is 10^3^ times higher compared to the cytosol concentration, and at pH = 6.5 (Figure 3b), this concentration is less than 10^1^ times higher compared to cytosol. This reveals that the lysosomal trapping is significantly lower at pH 6.5.

In addition to the lysosomal trapping evaluation in MembranePlus™, a parameter sensitivity analysis (PSA) was run in GastroPlus^®^, showing the influence of the fraction unbound in the enterocytes (F_uent_) in the T_max_ values.

**Figure 4:**
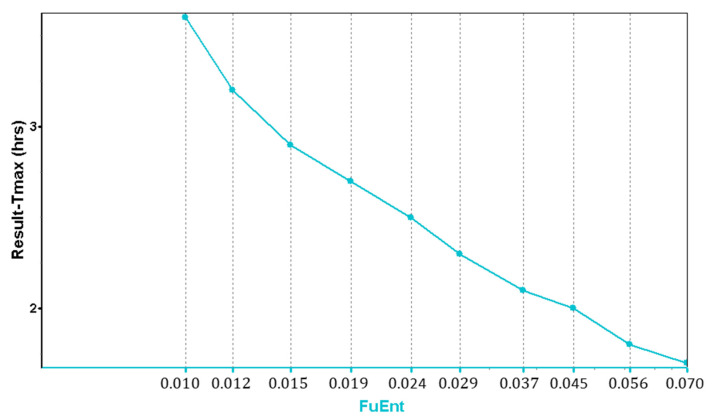
PSA showing the influence of the fraction of drug unbound in the enterocytes in the T_max_ values.

For IR tablets, the observed T_max_ values range from 0.5 to 6 h for the 50 mg dose [14], and from 2–4 h for the 100 mg dose [14]. Based on these values, and according to the PSA graph (Figure 4), a value of F_uent_ = 2% was used in the model for the simulations.

A two-compartment pharmacokinetic model was established using the IV and IR data as a top-down approach, in which values of the PK parameters are: T1/2 = 9.7 h, Cl = 0.2031 L/h/kg, Vss = 2.2343 L/kg, K12 = 0.15764 1/h, and K21 = 0.19244 1/h. These values are within the values described in the literature [13,14]. The values of the predicted versus observed PK parameters are shown in Table 4.

### 3.2. Statistical and In Silico Evaluation to Define Biopredictive Method

**Figure 5:**
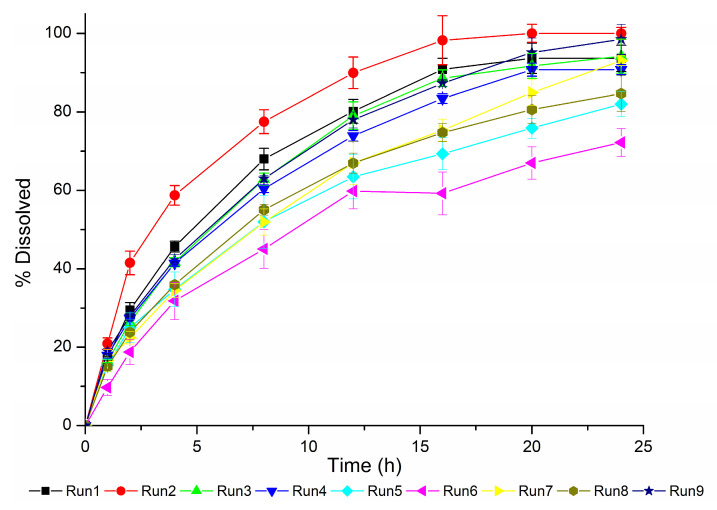
Dissolution profiles obtained using 900 mL of each dissolution medium at 37 °C and the test conditions described in the DoE (Table 3).

The dissolution profiles obtained based on the DoE are presented in Figure 5.

The DoE statistical analyses are presented in Tables S1–S3 (Supplementary Materials). These results revealed that the apparatus and formulation employed had a significant influence on the percentage of drug dissolved at all time points studied, as indicated by the *p*-value < 0.05. This finding suggests that the apparatus and formulation (drug product) are crucial factors affecting the dissolution of desvenlafaxine ER tablets. Conversely, the analysis did not demonstrate that the rotation speed had a significant impact on dissolution, as evidenced by the *p*-value > 0.05 for all studied responses.

The influence of the dissolution medium warrants further investigation. The Q%2 h and Q%24 h results indicate that the dissolution medium has an influence on drug dissolution (*p*-value < 0.05), despite the high solubility of desvenlafaxine in all studied dissolution media. However, this information conflicts with the finding that the dissolution medium had no effect on Q%12 h (*p*-value > 0.05). The discrepancy may be attributed to the indirect influence of other dissolution parameters on the result, particularly given the ultra-fractionated Taguchi experimental design employed.

This kind of design is defined by an orthogonal array, which is generally used for variable screening, allowing the evaluation of different factors performing a limited number of experiments [18]. Considering the adopted strategy (DoE + in silico model), it was decided to carry out the minimum amount of dissolution tests. As desvenlafaxine is a class I drug [4], we may infer that the dissolution medium does not significantly have influence on desvenlafaxine dissolution from the ER tablets analyzed.

Additionally, it is also worth mentioning that the Taguchi array is an experimental design that is already used in pharmaceutical technology to support the development of controlled release formulations, for example, in the evaluation of process and formulation variables in the development of a gastroretentive rivastigmine system [19], the development and optimization of solid lipid nanoparticles of isradipine [20], and in the production of carbamazepine [21] and cilostazol [22] osmotic pumps.

The average weight, thickness, and diameter/length of each drug product tablets are shown in Table 5. These results reveal that the SA/V ratio and weight of Generic #1 are, respectively, higher and lower than the other products analyzed. The higher SA/V ratio should favor the drug release since all tablets analyzed relies on a hypromellose (HPMC) hydrophilic matrix to extend the drug release, and a higher SA/V ratio, in these cases, is directly related to a faster dissolution profile [15]. The lower weight may also be indirectly influencing the drug release since it means that the HPMC is at a lower concentration in the final formulation, resulting in a less consistent gel after matrix hydration, and therefore favoring the drug release [23].

The dissolution profiles obtained (Figure 5) confirm this hypothesis as the Generic #1 profiles have the higher dissolution rates. The information that this product has a facilitated release is crucial to properly analyze the in silico results of the predictive errors obtained throughout convolution of the in vitro dissolution results for the DoE runs.

Table 6 contains the results of the C_max_, AUC_0-t_ and their respective prediction errors (PE%) obtained using Gastroplus^®^ to convolute the in vitro dissolution results (Figure 5) for each DoE run condition. A method was considered biopredictive for the evaluated drug product when both C_max_ and AUC_0-t_ predictive errors showed values within ±10% [24].

For the Generic #1 product, runs 1 and 3 are more biopredictive, based on the results obtained. These methods provide conditions that do not contribute to drug release from the matrix, since the method used in Run 1 consists of the use of basket apparatus at 50 rpm (Table 3), which promotes low hydrodynamics, and Run 3 does not use a sinker to hold the tablet during the assay, which implies in the adherence of the tablet in the bottom of the dissolution vessel. The Run 2 method, which is not biopredictive for Generic #1, is based on the use of paddle apparatus and sinkers to hold the tablets (Table 3). This condition promotes high hydrodynamics and prevents tablet adherence, fully hydrating the tablets by the end of the assay; therefore, it is a condition that facilitates drug release [25].

A different behavior was observed for Generic #2 and Reference products, compared to Generic #1, most likely due to the higher tablet weight and lower SA/V ratio (Table 5). In this case, the methods that promoted drug release (Run 4 and Run 9) were more biopredictive. At Run 4, the Reference product was evaluated using paddle at 100 rpm and sinkers (Table 3). This condition promotes high hydrodynamics and prevents tablet adherence, being a condition that facilitated drug release. Generic #2 was evaluated in Run 9, using the paddle apparatus at 50 rpm and sinkers (Table 3), conditions that prevent tablet adherence and promotes gentler agitation, compared to Run 4. The other methods studied would either use the basket apparatus that promotes low hydrodynamics or promotes tablet adherence by using the paddle apparatus with no sinker holding the tablets, thus not considered to be biopredictive [25].

**Figure 6:**
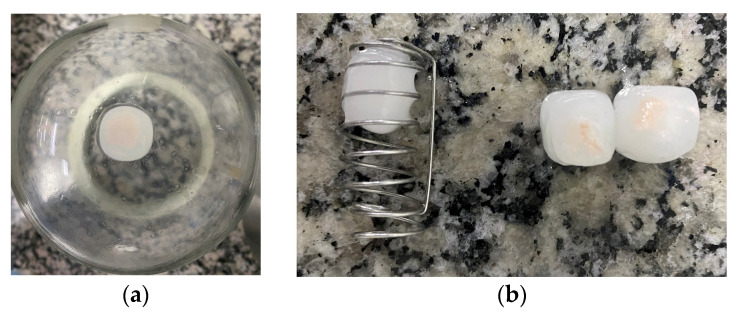
Images of the desvenlafaxine ER tablets after a 24 h dissolution assay, being: (**a**) the image of the tablet adhered to the bottom of the vessel after removing dissolution media; (**b**) image of the tablets after dissolution testing using sinkers, in the left, and with no use of sinkers, on the right.

Figure 6a,b show the Reference product tablets after a dissolution assay with and without the use of sinkers to hold the tablets. Figure 6a represents the table adhered to the bottom of the dissolution vessel. Figure 6b represents the difference in tablet gel formation and hydration after the assays, with the use of a sinker, in the left, and without this use, in the right. The same behavior was observed for all products studied.

These results show that, since Generic #1 have lower tablet weight and SA/V ratio, than Generic #2 and Reference products, for a method to be biopredictive for all drug products, it would have to promote drug release in Generic #2 and Reference, but also cannot promote such high hydrodynamics that would intensify drug release in Generic #1. In this case, the use of the basket apparatus may be discarded, since even with the higher rotation speed applied (Run 8), this condition is not biopredictive for Generic #2. In addition, the use of the paddle apparatus with no sinker holding the tablets is not a biopredictive condition, since the adherence of the tablets in the vessel bottom has a high impact on the matrix hydration, preventing the desvenlafaxine from being fully released from the Generic #2 and Reference tablets. Therefore, the most potential condition of being biopredictive is the paddle apparatus at 50 rpm and the use of sinkers. This condition promoted a relatively lower hydrodynamic in the vessel that would favor Generic #1 and would not inhibit drug release from Generic #2 and Reference tablets.

According to the previous discussion, the dissolution media do not have influence on the drug release. Thus, it was maintained as 0.9% NaCl, as suggested by the FDA for desvenlafaxine ER tablets [9]. In a recent study, Da Silva et al. (2020) [16] suggested the use of the basket apparatus at 75 rpm and a reduced volume of dissolution medium (500 mL) as a biorelevant method. However, according to the low hydrodynamics of the basket, as previously discussed, we decided to consider, as biopredictive, the following dissolution method: 900 mL of 0.9% NaCl and paddle at 50 rpm with sinker, which does not interfere with the performance of the matrix system.

### 3.3. In Vitro Dissolution Using Biopredictive Method and Virtual Bioequivalence Studies

**Figure 7:**
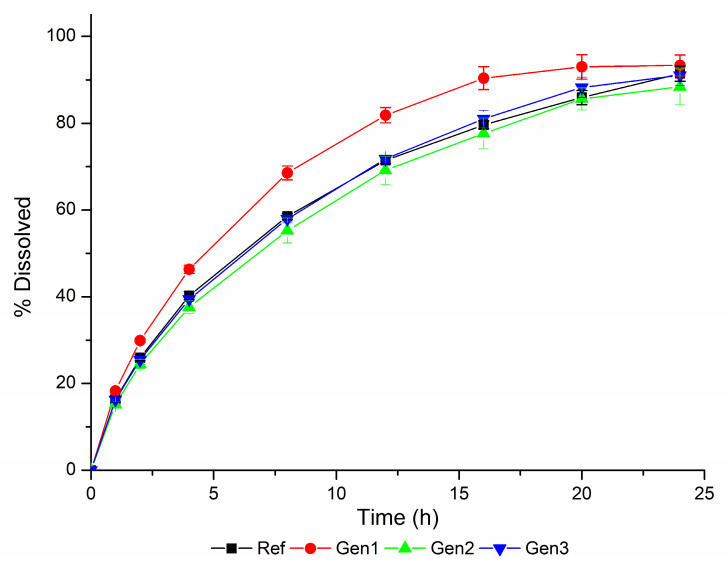
Dissolution profiles of the drug products Reference (Ref), Generic #1 (Gen 1), Generic #2 (Gen 2), and Generic #3 (Gen 3), evaluated using the biopredictive dissolution method.

The in vitro dissolution profiles obtained using the biopredictive dissolution method are shown in Figure 7.

The VBE studies of the drug products Generic #1, Generic #2, and Generic #3 versus Reference, using the dissolution profiles generated using the biopredictive dissolution method (Figure 7) led to the results presented in Tables S4–S6. Ten trials were performed for each VBE, and, in summary, the number of pass/fails are presented in Table 7.

Based on these results, we confirm that the dissolution method developed is biopredictive, since it is capable of detecting the potential of all Generic products studied to pass bioequivalence. The Generic #3, used as external validation, passed in all 10 VBE. The Generic #2 passed in 9 out of 10 VBE, which would be considered a good candidate for a Generic product. Lastly, Generic #1 passed in 7 out of 10 VBE, which is the majority of the tests and expresses the capability of the method to identify it as a good candidate; still, it shows that this product may be reproved on bioequivalence study depending on the individual characteristics of the participants on the bioequivalence study.

Although any datum from a non-bioequivalent ER desvenlafaxine formulation was used, and a dissolution/bioequivalence safe space was not defined, it was possible to obtain a biopredictive dissolution method. The ability of this method to detect non-bioequivalent products can be observed in Figure 7 and Table 7, in which, as the dissolution profile deviates from that of the reference drug product (Generic #1), the chance of failing bioequivalence increases.

Considering this behavior, we decided to set dissolution specifications based on the best results in virtual bioequivalence (Generic #2 and Generic #3) in addition to the reference drug product profile. Thus, a possible specification for desvenlafaxine ER tablets to ensure bioequivalence would be: 1 h (<20%), 4 h (38–48%), 8 h (50–72%), 16 h (72–93%), and 24 h (not less than 80%).

When comparing this specification with the one presented by Da Silva et al. (2020) [16], a difference can be observed, mainly at 4 h and 8 h, since the authors proposed a slightly lower range (4 h: 28–48%; 8 h: 48–68%), which may be attributed to the use of basket apparatus. It is worth mentioning that each specification is related to the dissolution method employed.

## 4. Conclusions

The integration between DoE and PBBM led to a rational development of a biopredictive dissolution method for desvenlafaxine ER tablets. This approach also provided an extensive understanding of the influences that dissolution test conditions have on desvenlafaxine in vitro and in vivo release, allowing for a reduced number of dissolution experiments to be performed. Finally, the knowledge generated with this study can help pharmaceutical scientists with drug product and dissolution method development.

## Figures and Tables

**Table 1:** Desvenlafaxine physicochemical and biopharmaceutical properties used as input data in GastroPlus^®^.

Parameters	Values	Source
pKa	10.45 (acid) and 9.18 (base)	[4]
logP	2.63	ADMET Predictor^®^
Molecular weight (g/mol)	263.38	ADMET Predictor^®^
Diffusion coefficient (cm/s × 10^−5^)	0.75	ADMET Predictor^®^
Peff (cm/s × 10^−4^)	6.2	[4]
B/P ^1^	1.08	ADMET Predictor^®^
Fup (%)	70	[4]
Solubility (mg/mL)	0.13 at pH 10.02	[4]

^1^ B/P: Blood-to-plasma concentration ratio.

**Table 2:** Taguchi 4-factor/3-level (L9) matrix design used to define the dissolution methods.

Factors	Levels		
	−1	0	1
Product	Generic #1	Reference	Generic #2
Dissolution medium	0.9% NaCl in purified water	0.05 M phosphate buffer pH 6.8	Purified water
Apparatus	Basket	Paddle with sinker	Paddle
Rotation speed	50 rpm	75 rpm	100 rpm

**Table 3:** Runs representing the dissolution methods obtained using the Taguchi design (L9).

Run	Product	Dissolution Medium	Apparatus	Rotation Speed
1	Generic #1	0.9% NaCl in purified water	Basket	50 rpm
2	Generic #1	0.05 M phosphate buffer pH 6.8	Paddle with sinker	75 rpm
3	Generic #1	Purified water	Paddle	100 rpm
4	Reference	0.9% NaCl in purified water	Paddle with sinker	100 rpm
5	Reference	0.05 M phosphate buffer pH 6.8	Paddle	50 rpm
6	Reference	Purified water	Basket	75 rpm
7	Generic #2	0.9% NaCl in purified water	Paddle	75 rpm
8	Generic #2	0.05 M phosphate buffer pH 6.8	Basket	100 rpm
9	Generic #2	Purified water	Paddle with sinker	50 rpm

**Table 4:** Predicted (Pred) and observed (Obs) PK parameters obtained for IR tablet 50 mg and IR tablet 100 mg. Observed data: IR tablet 50 mg [4,14] and IR tablet 100 mg [14].

Parameter	IR Tablet 50 mg			IR Tablet 100 mg		
	Obs	Pred	P/O	Obs	Pred	P/O
C_max_ (ng/mL)	249.5	256.8	1.03	532.0	513.48	0.97
T_max_ (h)	3.99	2.64	0.66	3.30	2.64	0.80
AUC_0-inf_ (ng-h/mL)	3617.8	3490.1	0.96	--	6988.4	--
AUC_0-t_ (ng-h/mL)	2931.0	2850.2	0.97	6251.0	5702.8	0.91

**Table 5:** Average weight, thickness, diameter/length, and surface area-per-volume ratio (SA/V ratio) of the drug products Reference, Generic #1, and Generic #2 tablets.

Drug Product	Average Weight (mg) ± SD	Thickness (cm) ± SD	Diameter (cm) ± SD	SA/V Ratio (cm^−1^) ± SD
Reference	362.0 ± 3.9	0.488 ± 0.005	0.913 ± 0.002	8.479 ± 0.049
Generic #1	210.0 ± 3.3	0.440 ± 0.005	0.818 ± 0.002	9.440 ± 0.058
Generic #2	362.0 ± 5.3	0.576 ± 0.009	0.920 ± 0.002	7.824 ± 0.052

**Table 6:** Results of C_max_ and AUC_0-t_ and the respective percent of prediction error (PE%) of the convolution results of the nine runs from DoE.

Run	C_max_ (ng/mL)			AUC_0-t_ (ng/mLxh)		
	Obs	Pred	PE%	Obs	Pred	PE%
Run 1 ^1^	77.23	83.48	−8.09	1866.80	1783.20	4.48
Run 2	77.23	96.84	−25.40	1866.80	1902.20	−1.90
Run 3 ^1^	77.23	79.35	−2.75	1866.80	1748.60	6.32
Run 4 ^1^	77.23	80.06	−3.67	1866.80	1738.70	6.86
Run 5	77.23	74.84	3.09	1866.80	1596.30	14.49
Run 6	77.23	67.77	12.25	1866.80	1493.20	20.01
Run 7	77.23	72.83	5.69	1866.80	1656.40	11.27
Run 8	77.23	74.50	3.52	1866.80	1633.80	12.48
Run 9 ^1^	77.23	81.13	−5.04	1866.80	1776.90	4.811

^1^ Runs 1, 3, 4, and 9 considered biopredictive.

**Table 7:** Number of pass/fails for each VBE performed using the dissolution profiles obtained using the biopredictive dissolution method.

VBE Performed	PASS	FAIL
Generic #1 vs. Reference	07	03
Generic #2 vs. Reference	09	01
Generic #3 vs. Reference	10	0

## Data Availability

The data presented in this study are available in the article and in the Supplementary Materials.

## Supplementary Materials

The following supporting information can be downloaded at: https://www.mdpi.com/article/10.3390/pharmaceutics15051544/s1, Table S1: Analysis of variance of Q%2 h; Table S2: Analysis of variance of Q%12 h; Table S3: Analysis of variance of Q%24 h; Table S4: Geometric mean, ratio between Generic 1 and Reference, and 90% CI of C_max_ and AUC_0-t_ from the virtual bioequivalence study using the biopredictive dissolution method, as a crossover study with 25 virtual subjects; Table S5: Geometric mean, ratio between Generic 2 and Reference, and 90% CI of C_max_ and AUC_0-t_ from the virtual bioequivalence study using the biopredictive dissolution method, as a crossover study with 25 virtual subjects; Table S6: Geometric mean, ratio between Generic 3 and Reference, and 90% CI of C_max_ and AUC_0-t_ from the virtual bioequivalence study using the biopredictive dissolution method, as a crossover study with 25 virtual subjects.
