# Supplementary material for: Development of Biopredictive Dissolution Method for Extended-Release Desvenlafaxine Tablets
Source: Pharmaceutics. 2023 May 19;15(5):1544. doi: 10.3390/pharmaceutics15051544 (PMC10222762; doi:10.3390/pharmaceutics15051544)
Supplement: Supplementary file 1 [file pharmaceutics-15-01544-s001.zip › pharmaceutics-2350580-supplementary.pdf]

Table S1. Analysis of variance of Q%2h

Effect	Sum of squares	Degrees of freedom	Average squares	F	p-value
(1) Drug product	412.5274	2	206.2637	43.19826	0.000000
(2) Dissolution medium	151.5696	2	75.7848	15.87178	0.000106
(3) Apparatus	381.5030	2	190.7515	39.94950	0.000000
(4) Rotation speed	17.4807	2	8.7404	1.83052	0.188947
Error	85.9467	18	4.7748		

Table S2. Analysis of variance of Q%12h

Effect	Sum of squares	Degrees of freedom	Average squares	F	p-value
(1) Drug product	1433.662	2	716.8311	48.81568	0.000000
(2) Dissolution medium	10.127	2	5.0633	0.34481	0.712933
(3) Apparatus	758.349	2	379.1744	25.82150	0.000005
(4) Rotation speed	11.642	2	5.8211	0.39641	0.678459
Error	264.32	18	14.6844		

Table S3. Analysis of variance of Q%24h

Effect	Sum of squares	Degrees of freedom	Average squares	F	p-value
(1) Drug product	1114.290	2	557.1448	50.92731	0.000000
(2) Dissolution medium	90.187	2	45.0937	4.12191	0.033590
(3) Apparatus	847.365	2	437.1826	39.96185	0.000000
(4) Rotation speed	20.661	2	10.3304	0.94428	0.407403
Error	196.92	18	10.9400		

Table S4.

Geometric mean, ratio between Generic 1 and Reference, and 90% CI of C_{max} and AUC_{0-t} from the virtual bioequivalence study using the biopredictive dissolution method, as a crossover study with 25 virtual subjects.

		Geometric Mean					Geometric Mean				
		Generic 1	Reference	Ratio	90% CI		Generic 1	Reference	Ratio	90% CI	
Trial #	PASS/FAIL	Cmax (µg/mL)	Cmax (µg/mL)	Cmax	Lower	Upper	AUCt (ng*hr/mL)	AUCt (ng*hr/mL)	AUCt	Lower	Upper
1	PASS	0.078	0.073	107.2	98.944	116.05	1755.2	1712.2	102.5	91.295	115.11
2	PASS	0.085	0.077	110.3	100.43	121.17	1984.0	1936.4	102.5	91.743	114.42
3	FAIL	0.090	0.081	111.0	98.492	125.14	2015.5	1931.1	104.4	95.374	114.21
4	PASS	0.087	0.077	112.6	102.32	123.86	1898.9	1756.7	108.1	98.163	119.02
5	PASS	0.091	0.080	114.1	105.64	123.33	1904.6	1723.1	110.5	99.516	122.77
6	PASS	0.081	0.072	112.1	100.98	124.44	1758.5	1667.8	105.4	92.596	120.06
7	PASS	0.085	0.078	108.0	98.093	118.88	1795.8	1759.9	102.0	88.959	117.04
8	FAIL	0.089	0.077	116.2	104.07	129.82	2008.8	1869.1	107.5	95.221	121.31
9	PASS	0.080	0.072	111.0	103.87	118.57	1808.4	1734.2	104.3	95.433	113.94
10	FAIL	0.079	0.069	114.1	102.19	127.37	1642.4	1526.7	107.6	94.781	122.1

Table S5.

Geometric mean, ratio between Generic 2 and Reference, and 90% CI of C_{max} and AUC_{0-t} from the virtual bioequivalence study using the biopredictive dissolution method, as a crossover study with 25 virtual subjects.

		Geometric Mean					Geometric Mean				
		Generic 2	Reference	Ratio	90% CI		Generic 2	Reference	Ratio	90% CI	
Trial #	PASS/FAIL	Cmax (µg/mL)	Cmax (µg/mL)	Cmax	Lower	Upper	AUCt (ng*hr/mL)	AUCt (ng*hr/mL)	AUCt	Lower	Upper
1	PASS	0.077	0.082	93.63	85.458	102.57	1832.5	1916.3	95.63	84.771	107.87
2	PASS	0.071	0.074	95.74	86.833	105.56	1763.4	1835.9	96.05	86.542	106.61
3	FAIL	0.078	0.088	88.05	79.896	97.044	1940.8	2109.5	92.00	82.768	102.27
4	PASS	0.073	0.073	100.9	91.551	111.25	1792.6	1751.8	102.3	90.500	115.71
5	PASS	0.075	0.078	96.73	89.845	104.14	1814.5	1775.8	102.2	94.133	110.92
6	PASS	0.070	0.073	95.62	85.956	106.36	1763.7	1803.9	97.77	86.828	110.1
7	PASS	0.076	0.078	97.92	88.287	108.61	1790.3	1752.5	102.2	92.578	112.73
8	PASS	0.077	0.078	97.86	88.048	108.77	1770.1	1772.8	99.85	87.561	113.86
9	PASS	0.076	0.079	96.01	88.53	104.13	1868.2	1859.4	100.5	90.209	111.9
10	PASS	0.076	0.077	98.94	90.768	107.84	1768.7	1719.4	102.9	92.428	114.48

Table S6.

Geometric mean, ratio between Generic 3 and Reference, and 90% CI of C_{max} and AUC_{0-t} from the virtual bioequivalence study using the biopredictive dissolution method, as a crossover study with 25 virtual subjects.

		Geometric Mean					Geometric Mean				
		Generic 3	Reference	Ratio	90% CI		Generic 3	Reference	Ratio	90% CI	
Trial #	PASS/FAIL	Cmax (µg/mL)	Cmax (µg/mL)	Cmax	Lower	Upper	AUCt (ng*hr/mL)	AUCt (ng*hr/mL)	AUCt	Lower	Upper
1	PASS	0.078	0.079	98.78	90.034	108.37	1918.0	1922.2	99.78	91.248	109.11
2	PASS	0.071	0.073	97.68	88.443	107.88	1692.3	1732.2	97.70	87.364	109.25
3	PASS	0.074	0.078	94.57	86.459	103.44	1778.2	1834.8	96.91	86.572	108.49
4	PASS	0.072	0.071	101.0	94.363	108.04	1704.6	1682.3	101.3	92.338	111.19
5	PASS	0.066	0.069	95.48	86.723	105.12	1499.1	1535.1	97.65	85.837	111.1
6	PASS	0.076	0.078	97.12	87.156	108.22	1898.2	1891.0	100.4	91.995	109.53
7	PASS	0.073	0.079	92.98	84.457	102.37	1649.8	1770.6	93.18	80.214	108.23
8	PASS	0.071	0.072	98.16	88.551	108.81	1717.4	1718.9	99.91	88.014	113.42
9	PASS	0.081	0.080	101.5	93.314	110.5	1977.3	1956.2	101.1	92.491	110.45
10	PASS	0.072	0.075	95.89	87.227	105.42	1771.7	1850.2	95.76	87.163	105.19
